# Non-Sexual Transmission of Papillomavirus: Is It Part of Our Virome?

**DOI:** 10.3390/v18080812

**Published:** 2026-07-24

**Authors:** Sandro La Vignera, Rosita A. Condorelli

**Affiliations:** Department of Clinical and Experimental Medicine, University of Catania, 95124 Catania, Italy; rosita.condorelli@unict.it

**Keywords:** human papillomavirus, non-sexual transmission, vertical transmission, virome, perinatal infection, fomite transmission, HPV prevalence, virgins

## Abstract

Background: Human papillomavirus (HPV) has traditionally been considered a sexually transmitted infection, yet accumulating evidence demonstrates that HPV can be acquired through multiple non-sexual routes. Understanding these alternative transmission pathways is critical for interpreting HPV detection in non-sexually active populations and for refining public health strategies. Methods: This review synthesizes current evidence on non-sexual HPV transmission routes, including vertical (transplacental, intrapartum), perinatal oropharyngeal colonization, fomite contamination, breast milk transmission, and horizontal non-sexual contact. We examine HPV prevalence data from female virgins, neonates, infants, and children, and evaluate metagenomic evidence positioning HPV as a component of the human virome across multiple body sites. Results: Published studies report that vertical transmission occurs in approximately 18.2% of HPV-positive mothers, with neonatal HPV positivity of 3.4% at birth and 100% genotype concordance in transmission pairs. Transplacental transmission has been documented in 10.2% of concordant mother–placenta–newborn triads. Reviewed studies report oropharyngeal colonization at birth reaching 58.2% following vaginal delivery, with 94.3% of colonized neonates clearing infection by 24 months. HPV DNA has been detected on fomites and medical devices, in breast milk (8.6–15%), and across body sites in healthy adults (skin 61.3%, vagina 41.5%, oral cavity 30%, gut 17.3%). Metagenomic studies identify HPV DNA in 68.9% of healthy individuals, with 109 distinct types detected. Female virgins show HPV prevalence ranging from 0–51.1% across studies. Conclusions: The reviewed evidence suggests that HPV exhibits characteristics of a ubiquitous virome component with multiple non-sexual acquisition routes. These findings have important implications for vaccination strategies, screening interpretation, infection control in healthcare settings, and counseling of pediatric cases and non-sexually active individuals.

## 1. Introduction

Human papillomavirus (HPV) represents one of the most prevalent viral infections worldwide, with over 200 identified genotypes infecting epithelial tissues at diverse anatomical sites. The dominant paradigm has long positioned HPV as a sexually transmitted infection (STI), with sexual contact serving as the primary route of acquisition for high-risk oncogenic types associated with cervical, oropharyngeal, and anogenital cancers. This framework has guided decades of public health policy, screening programs, and vaccination strategies targeting adolescents and young adults before sexual debut. However, an expanding body of evidence challenges the exclusivity of sexual transmission, revealing that HPV can be acquired through multiple non-sexual pathways throughout the human lifespan. The cumulative body of evidence for non-sexual HPV transmission has led to reconceptualization of HPV as a component of the normal human virome rather than exclusively a sexually transmitted pathogen [1,2,3].

The detection of HPV DNA in neonates born to HPV-positive mothers, in female virgins with no history of sexual contact, in young children with anogenital or laryngeal lesions, and across multiple body sites in healthy adults has prompted a fundamental re-evaluation of HPV epidemiology. Vertical transmission from mother to child—whether transplacental, intrapartum, or postpartum—has been documented with increasing frequency and molecular precision. Fomite contamination, environmental persistence of viral particles, and horizontal transmission through non-sexual close contact represent additional routes that may contribute to HPV acquisition independent of sexual activity. Furthermore, recent metagenomic surveys have revealed that HPV is a ubiquitous component of the human virome, with viral DNA detectable in the skin, oral cavity, gastrointestinal tract, and reproductive tissues of asymptomatic individuals at prevalences far exceeding those predicted by sexual transmission alone [4].

These findings carry profound implications for clinical practice and public health. The interpretation of HPV-positive test results in children, adolescents, and non-sexually active adults requires careful consideration of alternative acquisition routes to avoid inappropriate conclusions regarding sexual activity or abuse. Vaccination strategies may need to account for early-life exposures that precede sexual debut. Infection control protocols in healthcare settings must address the potential for nosocomial transmission via contaminated instruments and surfaces. Screening programs must distinguish between clinically significant persistent infections and transient colonization or low-level viral carriage that may represent components of the normal virome rather than pathogenic infections requiring intervention [1,5].

This review synthesizes current evidence on non-sexual HPV transmission routes, examines prevalence data from non-sexually active populations, evaluates the role of HPV as a component of the human virome, and discusses the clinical and public health implications of these findings. By integrating data from prospective cohort studies, systematic reviews, metagenomic surveys, and clinical case series, we aim to provide a comprehensive framework for understanding HPV epidemiology beyond the traditional sexual transmission paradigm and to identify critical knowledge gaps that warrant further investigation.

## 2. HPV Transmission Routes

### 2.1. Vertical and Perinatal Transmission

Vertical transmission of HPV from mother to child represents one of the most extensively documented non-sexual acquisition routes. This transmission can occur at multiple time points during pregnancy and delivery, including transplacental passage during gestation, intrapartum exposure during vaginal delivery, and postpartum transmission through breast milk or close contact. The molecular evidence for vertical transmission is compelling, with studies demonstrating genotype-specific concordance between maternal and neonatal HPV infections that cannot be explained by environmental contamination or independent acquisition.

**Figure 1:**
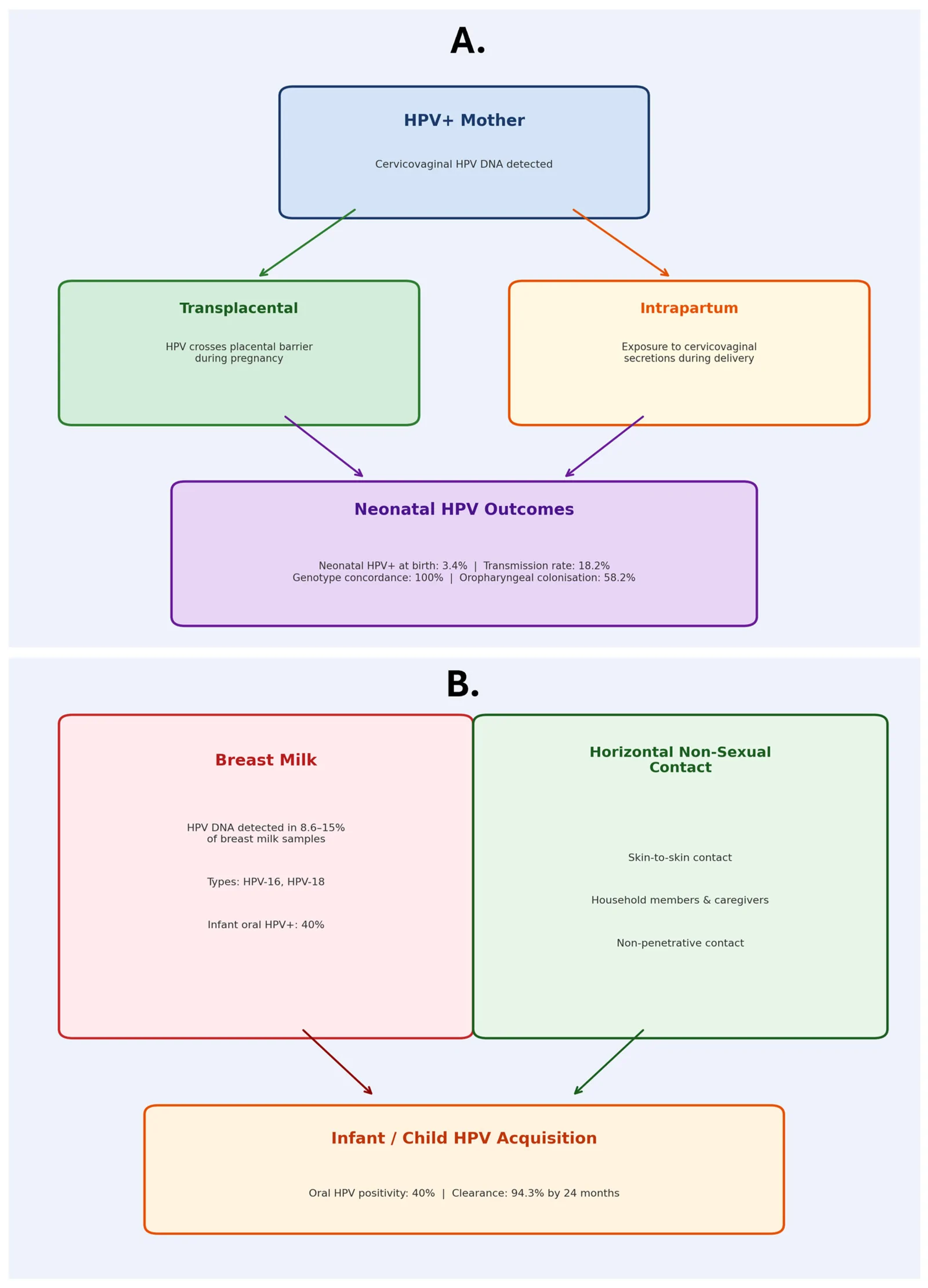

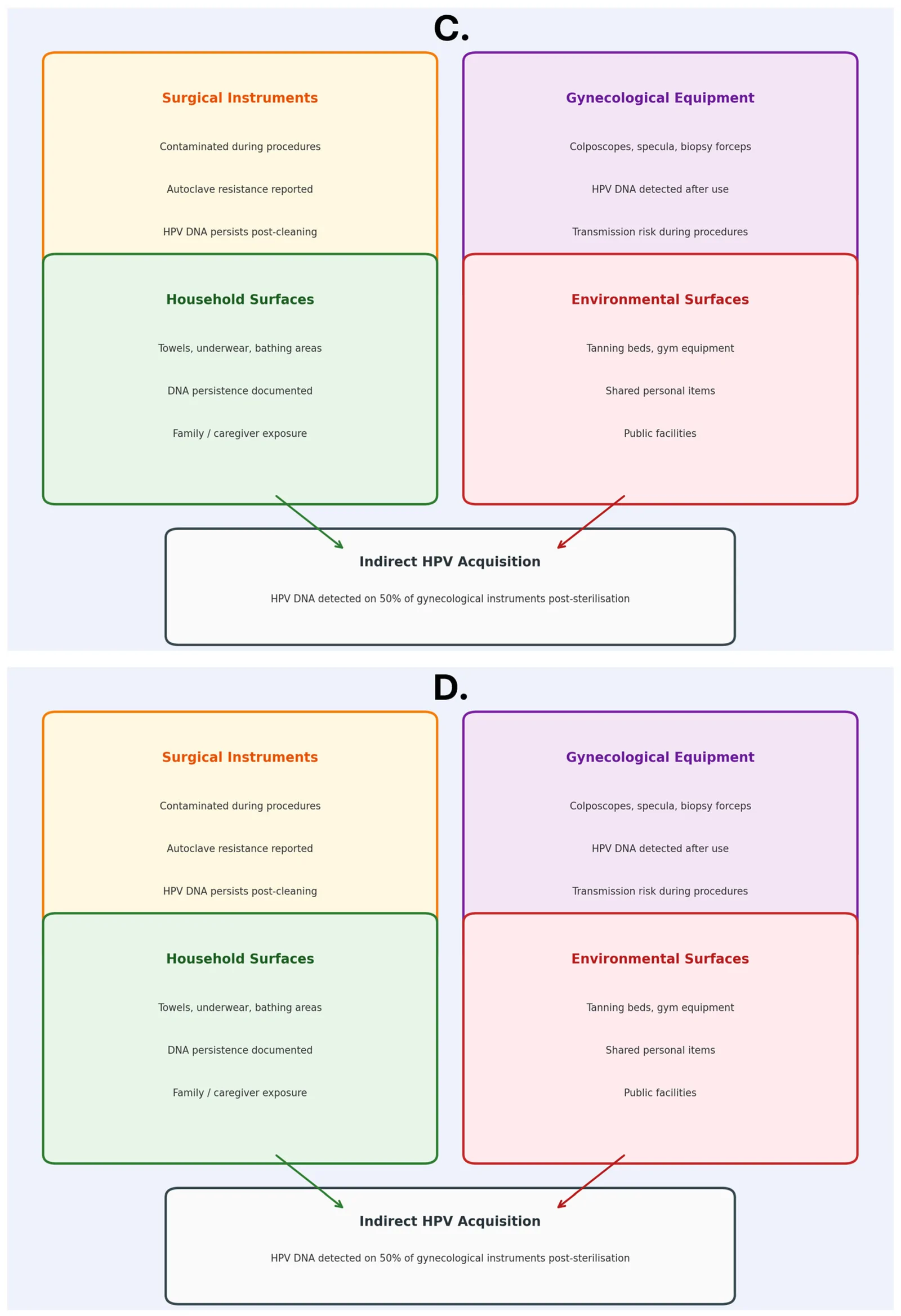
(**A**) Vertical Transmission of HPV. Schematic diagram illustrating the two vertical transmission routes: (1) transplacental—HPV crosses the placental barrier during pregnancy (concordance 10.2%); (2) intrapartum—neonatal exposure to cervicovaginal secretions during vaginal delivery. Both routes converge on documented neonatal HPV outcomes: positivity at birth 3.4%, transmission rate among HPV-positive mothers 18.2%, genotype concordance 100%, oropharyngeal colonization at birth 58.2%. (**B**) Postnatal HPV Transmission. Diagram showing two postnatal non-sexual acquisition routes: (1) breast milk—HPV DNA detected in 8.6–15% of samples, predominantly HPV-16 and HPV-18; infant oral HPV positivity 40%; (2) horizontal non-sexual contact—skin-to-skin contact, household members, and non-penetrative contact. Both routes contribute to infant/child HPV acquisition, with 94.3% spontaneous clearance by 24 months. (**C**) Fomite and Medical Device Transmission. Diagram illustrating four categories of indirect HPV transmission: (1) surgical instruments—HPV DNA persists post-cleaning, autoclave resistance documented; (2) gynecological equipment—colposcopes, specula, and biopsy forceps with post-use HPV DNA detection; (3) household surfaces—towels, underwear, and bathing areas; (4) environmental surfaces—tanning beds, gym equipment, and shared personal items. HPV DNA was detected on 50% of gynecological instruments after sterilization. (**D**) Respiratory and Salivary HPV Transmission. Diagram depicting two airborne/contact non-sexual routes from an HPV-positive individual: (1) salivary transmission—HPV DNA in saliva, kissing, and oral contact; oral HPV prevalence 30% in healthy adults; (2) respiratory droplets—aerosol spread, surgical smoke during ablative procedures, and association with recurrent respiratory papillomatosis (RRP). Both routes converge on clinical manifestations including RRP, oropharyngeal HPV carriage, and head and neck HPV-associated lesions.

A landmark prospective cohort study by Park and colleagues examined 291 mother–neonate pairs and detected HPV DNA in 18.9% of pregnant women and 3.4% of neonates at birth [1]. Among HPV-positive mothers, the vertical transmission rate was calculated at 18.2%, with 100% genotype concordance observed in all mother–neonate transmission pairs. This perfect concordance provides strong molecular evidence for true vertical transmission rather than coincidental infection with circulating genotypes. Importantly, all neonatal HPV detections in this cohort cleared spontaneously by 6 months of age, suggesting that many early-life infections represent transient colonization rather than persistent infection (Figure 1) [1].

The mechanisms underlying vertical transmission remain incompletely understood but likely involve multiple pathways. Intrapartum transmission during passage through an infected birth canal represents the most intuitive route, with neonatal exposure to maternal cervicovaginal secretions containing high viral loads. However, the detection of HPV DNA in placental tissue, amniotic fluid, and cord blood in some studies suggests that in utero transmission may also occur, potentially through hematogenous spread or ascending infection from the lower genital tract. The relative contributions of these different pathways likely vary depending on maternal viral load, genotype, immune status, and obstetric factors [1,6,7].

Mode of delivery appears to influence vertical transmission risk, with several studies reporting higher neonatal HPV detection rates following vaginal delivery compared to cesarean section. However, the detection of HPV in infants born by cesarean section with intact membranes indicates that intrapartum exposure is not the sole transmission route. Maternal HPV viral load at delivery correlates with transmission risk in some but not all studies, suggesting that host factors beyond simple viral burden may modulate transmission efficiency [8].

The clinical significance of vertical transmission remains a subject of ongoing investigation. While most neonatal infections appear to clear spontaneously within the first year of life, a subset of vertically transmitted infections persist and may lead to clinical disease. Juvenile-onset recurrent respiratory papillomatosis (JORRP), a rare but serious condition characterized by recurrent laryngeal papillomas caused by HPV types 6 and 11, is believed to result from vertical transmission in many cases. The factors determining whether a vertically transmitted infection clears spontaneously or progresses to persistent infection and disease remain poorly defined but likely involve viral genotype, inoculum size, anatomical site of infection, and host immune factors [1,6,8].

### 2.2. Transplacental Transmission

Transplacental transmission represents a particularly intriguing and controversial aspect of vertical HPV transmission. The detection of HPV DNA in placental tissue, amniotic fluid, and cord blood has been reported in multiple studies, suggesting that the virus can cross the placental barrier and potentially infect the fetus in utero. However, the frequency, mechanisms, and clinical significance of transplacental transmission remain subjects of active debate.

Rombaldi and colleagues conducted a detailed investigation of 49 HPV-positive pregnant women, examining maternal cervical samples, placental tissue, and neonatal specimens for HPV DNA [9]. Placental HPV DNA was detected in 24.5% of cases, indicating substantial viral presence in this tissue. Critically, type-specific concordance across the mother–placenta–newborn triad was observed in 10.2% of cases, providing molecular evidence for vertical transmission through the placental route. In one case (representing approximately 2% of the cohort), HPV DNA was detected in cord blood with genotype concordance to maternal and placental samples, strongly suggesting true transplacental transmission with fetal infection [2].

The mechanisms by which HPV might cross the placental barrier remain unclear. The placenta normally serves as an effective barrier against many pathogens through multiple mechanisms, including physical separation of maternal and fetal blood, immune surveillance, and expression of antiviral factors. However, several potential routes for HPV transplacental passage have been proposed. Direct infection of placental trophoblast cells could allow viral replication within the placenta and subsequent release into fetal circulation. Alternatively, cell-free viral particles or virus-infected maternal cells might cross the placental barrier through areas of inflammation, damage, or placental insufficiency. The detection of HPV DNA in placental tissue without corresponding fetal infection in many cases suggests that placental infection does not invariably lead to fetal transmission.

The genotypes detected in transplacental transmission studies span both high-risk and low-risk types. Rombaldi’s study identified multiple HPV types in placental and neonatal samples, including types 6, 11, 16, 18, 31, 33, 42, 52, and 58 [9]. The presence of high-risk oncogenic types in placental tissue raises questions about potential effects on pregnancy outcomes and fetal development, though definitive evidence linking HPV infection to adverse pregnancy outcomes remains limited and inconsistent across studies.

The clinical implications of transplacental transmission are uncertain. Unlike some other vertically transmitted infections (e.g., cytomegalovirus, rubella), HPV does not appear to cause a recognizable congenital infection syndrome with characteristic malformations or immediate neonatal disease. However, the long-term consequences of in utero HPV exposure remain largely unknown. Theoretical concerns include potential effects on fetal immune development, establishment of persistent infection in fetal tissues, and possible contributions to childhood malignancies, though evidence for these outcomes is currently lacking [9].

Since Rombaldi’s seminal 2008 study, subsequent investigations have continued to explore transplacental HPV transmission using improved molecular methodologies. A 2022 comprehensive review by Ardekani and colleagues examined HPV infection during pregnancy and childhood, corroborating evidence that HPV can be detected in placental tissues and cord blood, while emphasizing that true transplacental fetal infection remains an uncommon event whose frequency is difficult to quantify due to methodological heterogeneity across studies [5]. Complementing this, Koskimaa and colleagues documented that HPV DNA presence in the placenta and/or cord blood may elicit T-cell mediated immune responses in the neonate, suggesting that in utero HPV exposure carries immunological consequences that may influence viral clearance or persistence during early life [8]. These findings highlight the need for prospective cohort studies with standardized molecular assays to accurately quantify the risk of transplacental transmission and its clinical implications, and to determine whether early immune priming by placental HPV exposure has downstream effects on HPV-related disease risk.

### 2.3. Intrapartum Oropharyngeal Colonization

Oropharyngeal colonization of neonates during vaginal delivery represents one of the most frequently documented forms of vertical HPV transmission. The neonate’s passage through an HPV-infected birth canal provides direct exposure to high concentrations of viral particles in cervicovaginal secretions, with potential for viral deposition on oral, nasal, and pharyngeal mucosa. Longitudinal studies tracking neonatal oropharyngeal HPV status from birth through early childhood have provided valuable insights into the natural history of these infections.

Hijona Elosegui and colleagues conducted a prospective study of neonates born vaginally to HPV-positive mothers, documenting remarkably high rates of oropharyngeal colonization at birth [10]. Among neonates delivered vaginally to HPV-positive mothers, 58.2% tested positive for oropharyngeal HPV DNA immediately after birth. This high colonization rate demonstrates the efficiency of intrapartum viral transmission to the neonatal oropharynx during vaginal delivery. However, the natural history of these infections proved to be predominantly transient, with 94.3% of colonized neonates clearing the virus by 24 months of age. Only 5.7% of initially colonized infants maintained persistent oropharyngeal HPV detection at 24 months [3].

These findings have important implications for understanding the clinical significance of neonatal HPV detection. The high initial colonization rate followed by near-universal clearance suggests that most intrapartum HPV exposures represent transient mucosal contamination or brief colonization rather than true persistent infection. The distinction between transient colonization and persistent infection is critical for clinical management and counseling. Transient detection likely reflects viral deposition on mucosal surfaces without productive infection of basal epithelial cells, whereas persistent detection may indicate true infection with viral replication and potential for disease progression.

The small subset of infants with persistent oropharyngeal HPV detection (5.7% in the Hijona Elosegui study) represents a population of particular clinical interest [10]. These persistently infected infants may be at risk for developing juvenile-onset recurrent respiratory papillomatosis (JORRP), a rare but serious condition characterized by recurrent growth of papillomas in the larynx and airways. JORRP is most commonly caused by HPV types 6 and 11 and typically manifests in early childhood with hoarseness, stridor, and respiratory distress. The condition requires repeated surgical interventions to remove papillomas and maintain airway patency, with significant morbidity and occasional mortality. JORRP represents the most significant clinical sequela of vertical HPV transmission. Recent epidemiological data from the United States indicate an incidence of approximately 1.7–2.6 per 100,000 children, with a mean age of onset between 3 and 4 years [11]. The incidence of JORRP has been declining in countries with high HPV vaccination coverage, providing compelling evidence that immunization before reproductive age can reduce the burden of perinatal HPV 6/11 transmission and its clinical sequelae in offspring [12,13]. HPV types 6 and 11 account for the vast majority of JORRP cases; type 11 is associated with more aggressive disease, higher recurrence rates, and a greater risk of distal airway spread to the trachea, bronchi, and lungs [14]. Management requires repeated surgical debulking under general anesthesia; adjuvant therapies including bevacizumab, cidofovir, and—more recently—the HPV prophylactic vaccine have been employed with variable success in reducing recurrence frequency [13]. A 2024 systematic review by Lépine and colleagues underscored that, despite decades of research, no curative therapy currently exists, and many patients undergo dozens of procedures over their lifetime, with substantial morbidity, cumulative anesthetic exposure, voice impairment, and in severe cases, tracheostomy dependency [15]. The potential role of HPV sublineage variation in influencing JORRP phenotype and severity is an emerging area of investigation [16]. The substantial disease burden and lack of curative options reinforce the critical importance of preventive strategies targeting vertical HPV transmission.

The factors determining which neonates clear oropharyngeal HPV colonization and which develop persistent infection remain incompletely understood. Potential determinants include viral genotype (with types 6 and 11 showing greater propensity for respiratory tract infection), viral load at initial exposure, anatomical site of viral deposition, and host immune factors. Maternal immune status and the transfer of protective antibodies through placental passage and breast milk may also influence neonatal clearance rates. Further research is needed to identify biomarkers that can predict which neonates with initial HPV detection are at risk for persistent infection and disease progression, enabling targeted surveillance and early intervention [10].

### 2.4. Fomites and Medical Devices

The environmental stability of HPV and its detection on inanimate surfaces and medical devices represents an underappreciated transmission route with important implications for infection control in healthcare and community settings. Unlike many enveloped viruses that are rapidly inactivated outside the host, HPV’s non-enveloped capsid structure confers substantial environmental stability, allowing viral particles to remain infectious on surfaces for extended periods.

Ryndock and Meyers conducted a comprehensive review of HPV environmental contamination and fomite transmission, documenting HPV DNA detection on a wide range of surfaces and objects [17]. HPV DNA has been identified on medical instruments including vaginal specula, colposcopes, and examination tables in gynecology clinics, even after standard cleaning procedures. In public settings, HPV DNA has been detected on surfaces in communal bathing facilities, public restrooms, and shared equipment. The virus can survive on dry surfaces for hours to days, depending on environmental conditions such as temperature, humidity, and UV exposure [4].

The detection of HPV DNA on medical devices raises significant concerns about nosocomial transmission. Sabeena and colleagues conducted systematic investigations of HPV contamination in healthcare settings, identifying viral DNA on reusable medical instruments and environmental surfaces in clinics performing gynecological examinations and procedures [18]. Standard cleaning and disinfection protocols used in many healthcare facilities may be inadequate for complete HPV inactivation, particularly for instruments with complex geometries or porous materials that can harbor viral particles. The potential for patient-to-patient transmission via inadequately disinfected instruments represents a serious infection control challenge [5].

Experimental studies have demonstrated that HPV can be transferred from contaminated surfaces to skin and mucosa through direct contact. Hand-to-genital transmission has been documented in studies of couples and individuals, with HPV DNA detected on fingers and under fingernails in sexually active individuals. This finding suggests that self-inoculation or partner-to-partner transmission can occur through manual contact, independent of penetrative sexual activity. In healthcare settings, transmission from contaminated gloves or instruments to patient mucosa represents a plausible route for nosocomial acquisition.

The clinical significance of fomite transmission remains difficult to quantify. While HPV DNA detection on surfaces confirms environmental contamination, the relationship between DNA detection and infectious viral particles capable of establishing infection is uncertain. PCR-based detection methods identify viral DNA but do not distinguish between intact infectious virions and degraded non-infectious viral fragments. Nevertheless, the documented cases of HPV infection in individuals with no apparent sexual contact or vertical transmission history suggest that environmental and fomite transmission may contribute to HPV epidemiology more substantially than previously recognized.

Ryndock and Meyers, along with Sabeena and colleagues, have called for enhanced infection control measures in healthcare settings to minimize fomite transmission risk [17,18]. Recommendations include use of disposable single-use instruments where feasible, implementation of high-level disinfection protocols for reusable instruments, regular environmental surface disinfection in examination rooms, and strict hand hygiene practices. For instruments that cannot be adequately disinfected, such as certain colposcopes and examination equipment, barrier methods (e.g., disposable covers) should be employed. Public health education about potential fomite transmission may also be warranted, particularly regarding shared bathing facilities and personal hygiene items [17,18].

### 2.5. Breast Milk

The detection of HPV DNA in breast milk represents a relatively recent discovery with important implications for understanding postnatal vertical transmission routes. Breast milk serves as a major route of viral transmission for several pathogens (e.g., HIV, cytomegalovirus, HTLV), and the identification of HPV in milk samples raises questions about the potential for oral transmission to nursing infants and the clinical significance of such exposures.

Glenn and colleagues conducted one of the first systematic investigations of HPV in breast milk, using next-generation sequencing to detect viral DNA in samples from lactating women [19]. HPV DNA was identified in 15% (6/40) of breast milk samples, with high-risk oncogenic types HPV-16 and HPV-18 detected in several cases. The presence of these high-risk genotypes in breast milk is particularly noteworthy given their strong association with cervical cancer and oropharyngeal malignancies. The mechanisms by which HPV enters breast milk remain unclear but may involve viral shedding from infected ductal epithelium, hematogenous spread with viral entry into milk, or contamination from skin lesions on the breast [11].

Tuominen and colleagues conducted complementary research examining HPV in family cohorts, detecting HPV DNA in 8.6% (3/35) of breast milk samples [20]. Importantly, this study also examined oral samples from nursing infants, finding HPV DNA in 40% (14/35) of infant oral samples. While the study design did not definitively establish breast milk as the source of infant oral HPV, the temporal relationship and family clustering suggest that breastfeeding may represent a route of postnatal HPV transmission. The high prevalence of oral HPV in nursing infants compared to the lower prevalence in breast milk suggests that additional transmission routes (e.g., maternal skin contact, environmental exposure) likely contribute to infant oral HPV acquisition [12].

The clinical significance of HPV transmission through breast milk remains uncertain. Unlike vertical transmission during delivery, which has been linked to juvenile-onset recurrent respiratory papillomatosis in a subset of cases, no specific disease syndrome has been definitively attributed to HPV acquisition through breastfeeding. The oral cavity and oropharynx of infants are exposed to HPV through breast milk, but whether this exposure leads to persistent infection, transient colonization, or immune priming without infection is unknown. Longitudinal studies tracking infants from birth through early childhood, with serial sampling of breast milk and infant oral specimens, are needed to clarify the natural history of breast milk-associated HPV exposures.

The detection of high-risk HPV types in breast milk also raises theoretical concerns about potential long-term consequences. HPV-16 and HPV-18 are the primary causative agents of oropharyngeal squamous cell carcinoma in adults, a malignancy that has increased dramatically in incidence in recent decades. Whether early-life oral exposure to these high-risk types through breast milk influences later risk of oropharyngeal cancer is purely speculative at present, but represents an important question for future epidemiological research.

Current public health guidance does not recommend against breastfeeding for HPV-positive mothers. The well-established benefits of breastfeeding for infant health, immune development, and maternal–infant bonding far outweigh the theoretical risks associated with HPV transmission through milk. However, the findings of Glenn, Tuominen, and colleagues underscore the need for continued research into the prevalence, mechanisms, and consequences of HPV in breast milk [19,20].

### 2.6. Respiratory and Horizontal Non-Sexual Contact

Respiratory transmission and horizontal non-sexual contact represent additional routes through which HPV may be acquired, particularly in childhood and in household settings. While less well-characterized than vertical transmission or fomite contamination, accumulating evidence suggests that close non-sexual contact between individuals can facilitate HPV transmission through mechanisms that do not involve sexual activity.

Sinclair and colleagues conducted a comprehensive review of HPV infections in children, identifying 124 pediatric cases with HPV-associated lesions including laryngeal papillomas, anogenital warts, and oral lesions [21]. The mean age at diagnosis was approximately 4 years, with many cases occurring in prepubertal children with no possibility of sexual transmission. The authors concluded that many prepubertal anogenital and laryngeal HPV infections are likely acquired perinatally or through non-sexual contact, with sexual abuse representing a more likely explanation in older children with anogenital lesions. The presence of laryngeal papillomatosis in young children is particularly indicative of perinatal transmission, as this condition typically results from vertical transmission of HPV types 6 and 11 during delivery [7].

Horizontal transmission within households has been documented in several studies. HPV DNA has been detected on the hands and under the fingernails of family members, suggesting that hand-to-mucosa transmission may occur through routine caregiving activities, shared bathing, or other close contact. In couples, hand-to-genital and genital-to-hand transmission has been demonstrated, indicating that manual contact can transfer infectious viral particles. In pediatric populations, similar mechanisms may facilitate transmission from parents to children or between siblings through activities such as bathing, diaper changing, or shared use of towels and clothing.

Respiratory transmission of HPV remains controversial and poorly documented. Unlike respiratory viruses that are efficiently transmitted through aerosols or respiratory droplets, HPV is not typically considered a respiratory pathogen. However, the detection of HPV DNA in saliva, oral rinses, and respiratory secretions raises the theoretical possibility of transmission through close contact involving respiratory secretions. Kissing, sharing of eating utensils, and close face-to-face contact could potentially transmit oral HPV, though the efficiency of such transmission and its contribution to overall HPV epidemiology remain uncertain.

The distinction between sexual and non-sexual transmission becomes particularly important in pediatric cases of anogenital HPV infection. The detection of anogenital HPV in a child has historically raised concerns about sexual abuse, and HPV infection has been used as a marker of abuse in forensic evaluations. However, the recognition that anogenital HPV can be acquired through vertical transmission, fomite contact, or horizontal non-sexual transmission complicates this interpretation. Sinclair and colleagues emphasize that HPV detection alone cannot definitively establish sexual abuse, and that clinical evaluation must consider the child’s age, the anatomical site and type of lesion, the HPV genotype detected, and the overall clinical context [21].

**Figure 2:**
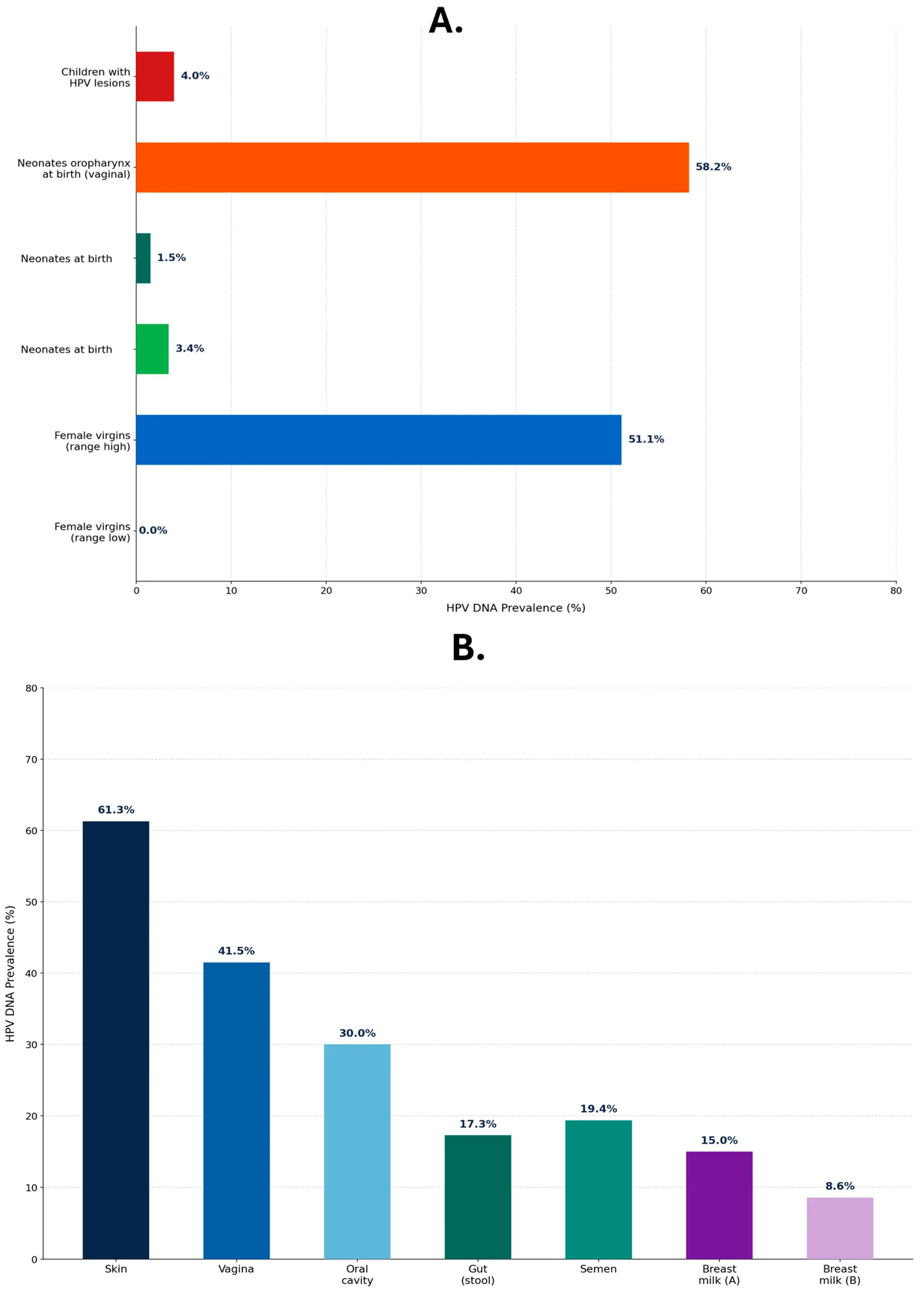

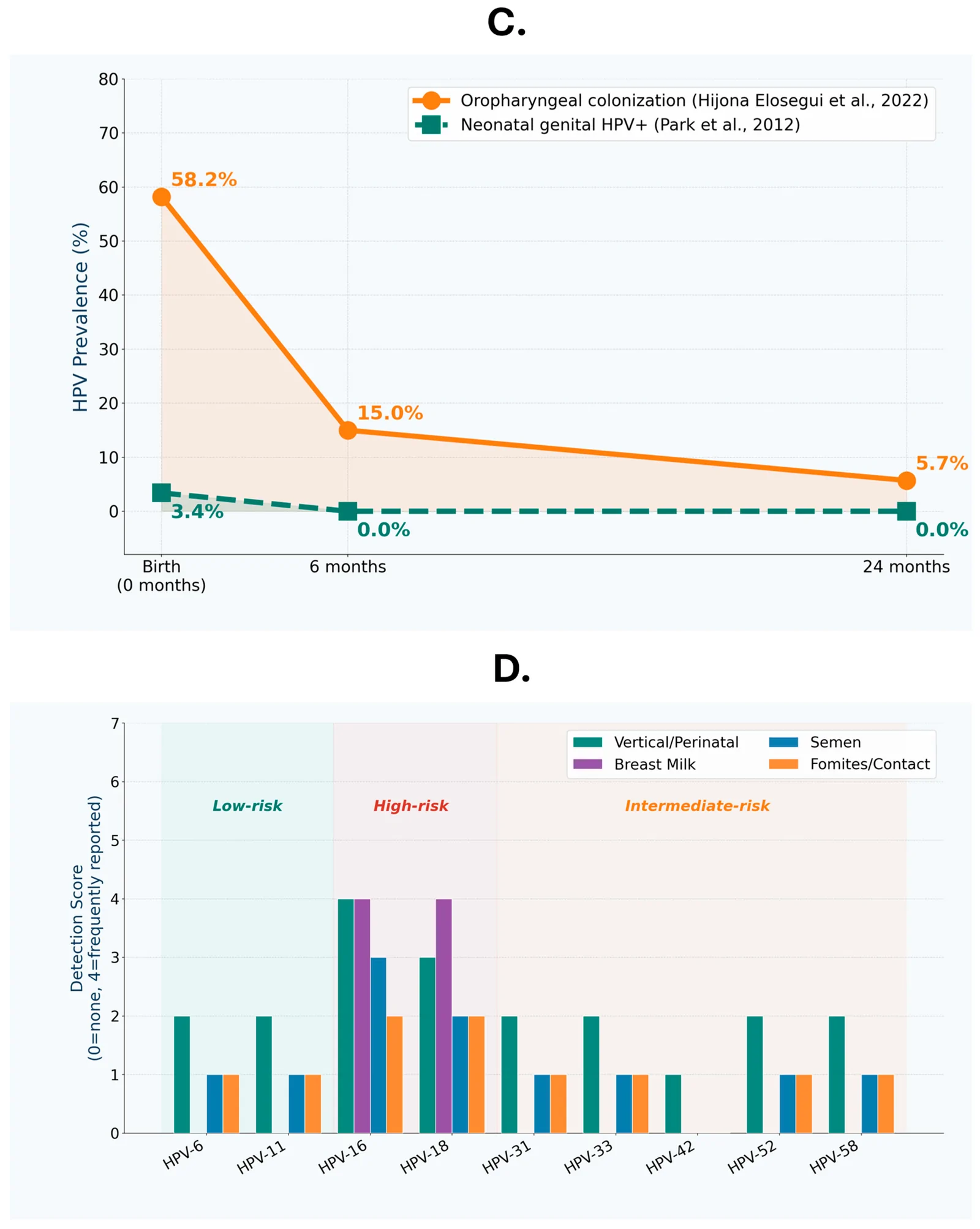
(**A**) HPV prevalence in non-sexually active groups and non-sexual bodily fluids. Horizontal bar chart comparing HPV detection rates across non-sexually active cohorts and non-sexual bodily fluids, including female virgins (0–51.1%), neonates at birth (3.4%), breast milk (8.6–15%), semen (19.4%), and metagenomic detection in healthy adults (68.9%). (**B**) Site-specific HPV prevalence in healthy adults (metagenomic data). Vertical bar chart illustrating the distribution of HPV across body sites in healthy individuals, based on metagenomic analysis: skin (61.3%), vagina (41.5%), oral cavity (30%), gut (17.3%), semen (19.4%), and breast milk (8.6–15%). Data from. (**C**) Neonatal HPV clearance over time. Line chart showing the temporal dynamics of neonatal HPV colonization following birth: oropharyngeal colonization declines from 58.2% at birth to 15.0% at 6 months and 5.7% at 24 months ([3]); neonatal genital HPV positivity (3.4% at birth) resolves completely by 6 months ([1]). (**D**) HPV genotypes detected by non-sexual transmission context. Grouped bar chart displaying detection scores (0–4 scale: 0 = not reported, 4 = frequently reported) for key HPV genotypes (HPV-6, -11, -16, -18, -31, -33, -42, -52, -58) across four non-sexual transmission contexts: vertical/perinatal, breast milk, semen, and fomites/contact. High-risk types HPV-16 and HPV-18 dominate across all contexts.

Sabeena and colleagues have called for enhanced research into non-sexual transmission routes, noting that current understanding remains limited by methodological challenges [18]. Distinguishing between different transmission routes in observational studies is difficult, as individuals may be exposed through multiple pathways simultaneously. Prospective cohort studies with detailed exposure assessment, frequent serial sampling, and molecular genotyping are needed to clarify the relative contributions of vertical, fomite, and horizontal transmission to HPV epidemiology across different age groups and populations (Figure 2) [5].

## 3. HPV Prevalence in Non-Sexually Active Individuals

### 3.1. Female Virgins

The detection of HPV DNA in female virgins represents one of the most compelling lines of evidence for non-sexual transmission. By definition, individuals with no history of sexual contact should not harbor sexually transmitted infections, yet multiple studies have documented HPV prevalence in virgin populations at rates that cannot be explained by false reporting of sexual history alone.

Liu and colleagues conducted a systematic review and meta-analysis of HPV prevalence in female virgins, synthesizing data from multiple studies across diverse populations [18]. The reported prevalence of genital HPV DNA in female virgins ranged from 0% to 51.1% across studies, reflecting substantial heterogeneity in study populations, sampling methods, and detection techniques. This wide range underscores the methodological challenges in studying HPV in virgin populations, including variations in the definition of virginity, differences in sampling sites (vulvar, vaginal, cervical), and differences in assay sensitivity and specificity [5].

Several factors contribute to the observed heterogeneity in HPV prevalence among virgins. Studies using more sensitive PCR-based detection methods generally report higher prevalence than those using less sensitive techniques. Sampling site also influences detection rates, with vulvar and perineal sampling potentially capturing HPV from skin or environmental contamination, while cervical sampling in virgins raises anatomical and ethical challenges. The definition of virginity varies across studies, with some defining it as no history of penetrative vaginal intercourse, while others include any form of sexual contact. Individuals reporting virginity but engaging in non-penetrative sexual activities (e.g., genital touching, oral-genital contact) may acquire HPV through these routes.

Despite these methodological challenges, the consistent detection of HPV DNA in at least some virgins across multiple independent studies provides strong evidence for non-sexual acquisition. Potential routes of HPV acquisition in virgins include vertical transmission (with persistence from perinatal infection), fomite transmission (from contaminated surfaces or objects), horizontal transmission (from close non-sexual contact with family members), and autoinoculation (from hand-to-genital transfer of virus from other body sites).

The clinical significance of HPV detection in virgins remains uncertain. Most HPV infections in virgins appear to involve low viral loads and may represent transient colonization or contamination rather than true persistent infection. The natural history of these infections—including rates of persistence, progression to dysplasia, and clearance—has not been well-characterized in virgin populations. Longitudinal studies following virgins from adolescence through sexual debut and beyond are needed to determine whether HPV detected before sexual activity influences subsequent infection dynamics and disease risk.

The detection of HPV in virgins has important implications for screening and vaccination strategies. Current cervical cancer screening guidelines generally do not recommend screening before age 21 or before sexual debut, based on the assumption that HPV is sexually transmitted and that cervical cancer is rare in young women. However, if non-sexual HPV acquisition is common, earlier screening or alternative screening strategies might be warranted for certain populations. Similarly, vaccination programs targeting pre-adolescent girls before sexual debut may provide protection not only against future sexual acquisition but also against non-sexual exposures that occur throughout childhood and adolescence [18].

### 3.2. Neonates and Infants

Neonatal and infant HPV prevalence data provide critical insights into vertical transmission rates and the natural history of early-life HPV infections. Studies examining HPV status at birth and during the first years of life have documented both the frequency of vertical transmission and the typical trajectory of neonatal infections.

As previously discussed, Park and colleagues documented HPV DNA in 3.4% (10/291) of neonates at birth in a prospective cohort study [1]. Among HPV-positive mothers, the vertical transmission rate was 18.2%, with perfect genotype concordance between mothers and infected neonates. Critically, all neonatal HPV detections in this cohort cleared spontaneously by 6 months of age, with no infants testing positive at the 6-month follow-up. This finding suggests that most vertically transmitted infections represent transient colonization that resolves without intervention [1].

Smith and colleagues conducted a complementary study examining a larger cohort of mother–newborn pairs [6]. In this study, 30% of mothers tested HPV-positive during pregnancy, but only 1.5% of newborns were HPV DNA-positive at birth. Among HPV-positive mothers, only 3% of their infants tested positive for HPV DNA. Importantly, genotype concordance between mothers and infants was rare, with only one mother–infant pair sharing the same HPV type. This lower transmission rate and reduced concordance compared to the Park study may reflect differences in sampling methods, timing of sample collection, or population characteristics [9].

The discrepancy between studies in reported vertical transmission rates highlights the methodological challenges in this research area. Factors influencing reported transmission rates include the timing of neonatal sampling (immediately after birth versus hours or days later), the anatomical site sampled (oral, nasopharyngeal, genital), the sensitivity of the detection method, and the definition of HPV positivity (any HPV DNA detection versus type-specific concordance with maternal infection). Studies sampling immediately after birth may detect transient contamination from maternal secretions, while studies sampling days later may miss transient colonization that clears rapidly.

The natural history of neonatal HPV infections appears to be predominantly one of spontaneous clearance. The Park study’s finding of 100% clearance by 6 months, combined with the Hijona Elosegui study’s documentation of 94.3% clearance of oropharyngeal colonization by 24 months, suggests that the neonatal immune system is generally effective at clearing HPV infections acquired at birth [1,10]. However, the small subset of infants with persistent detection represents a population at risk for clinical disease, particularly juvenile-onset recurrent respiratory papillomatosis.

The factors determining clearance versus persistence of neonatal HPV infections remain poorly understood. Potential determinants include viral genotype (with types 6 and 11 showing greater propensity for persistence and disease), viral load at initial infection, anatomical site of infection, and host immune factors. Maternal antibodies transferred transplacentally and through breast milk may provide passive immunity that facilitates viral clearance in most infants. Infants with immune deficiencies or those who fail to mount effective cellular immune responses may be at higher risk for persistent infection.

The clinical management of HPV-positive neonates remains controversial. Given the high rate of spontaneous clearance, routine screening of neonates for HPV is not recommended, and detection of HPV DNA in an asymptomatic neonate does not warrant intervention. However, infants born to mothers with active genital HPV infection, particularly with types 6 and 11, may warrant clinical surveillance for signs of respiratory papillomatosis, including monitoring for hoarseness, stridor, or respiratory distress during the first years of life [1,6,10].

### 3.3. Children

HPV infections in prepubertal children provide additional evidence for non-sexual transmission and raise important clinical and forensic questions. The detection of HPV in children, particularly at anogenital sites, has historically raised concerns about sexual abuse, but accumulating evidence indicates that many pediatric HPV infections result from non-sexual transmission routes.

Sinclair and colleagues conducted a comprehensive review of HPV infections in children, identifying 124 pediatric cases with HPV-associated lesions [21]. The mean age at diagnosis was approximately 4.0 years, with cases spanning from infancy through adolescence. The anatomical distribution of lesions included laryngeal papillomas, anogenital warts, and oral lesions. The authors analyzed the likely transmission routes for these infections based on age, lesion characteristics, and clinical context [7].

For laryngeal papillomatosis in young children, vertical transmission during delivery represents the most likely acquisition route. Juvenile-onset recurrent respiratory papillomatosis (JORRP) typically manifests in the first few years of life and is caused predominantly by HPV types 6 and 11. The temporal relationship between birth and disease onset, combined with the known presence of these types in maternal genital infections, strongly supports perinatal transmission. Mothers of children with JORRP frequently have a history of genital warts or HPV infection during pregnancy, though not all cases have documented maternal infection. As discussed in detail in Section 2.3, JORRP is the principal clinical manifestation of persistent perinatal HPV infection. Population-based studies confirm that JORRP incidence peaks between ages 2 and 4 years and is higher among children born vaginally to mothers with active condylomata acuminata at delivery [11,12]. Notably, only a small fraction of children born to HPV 6/11-positive mothers ultimately develop JORRP (estimated lifetime risk approximately 1 in 400), indicating that exposure alone is insufficient and that host immune factors play a critical modulating role [15]. Readers are directed to Section 2.3 for a comprehensive discussion of JORRP epidemiology, natural history, management, and prevention.

For anogenital HPV lesions in prepubertal children, the interpretation is more complex. Sinclair and colleagues concluded that in young children (particularly those under age 3), perinatal transmission or non-sexual horizontal transmission represents the most likely explanation [21]. Anogenital warts in infants and toddlers may result from vertical transmission with delayed clinical manifestation, autoinoculation from oral or cutaneous HPV, or transmission through routine caregiving activities. However, in older prepubertal children (particularly those over age 4–5), the possibility of sexual abuse must be considered, and anogenital HPV detection should prompt careful clinical evaluation including assessment for other signs of abuse, behavioral changes, and disclosure [21].

The genotypes detected in pediatric HPV infections span both low-risk and high-risk types. Types 6 and 11 predominate in laryngeal papillomatosis and many cases of anogenital warts, consistent with vertical transmission from maternal genital infections. However, high-risk types including HPV-16 and HPV-18 have also been detected in some pediatric cases, raising questions about the natural history and cancer risk associated with these infections in children.

The clinical management of pediatric HPV infections depends on the anatomical site and clinical presentation. Laryngeal papillomatosis requires surgical intervention to maintain airway patency, often with multiple procedures over years. Anogenital warts may be treated with topical therapies or surgical removal, though spontaneous regression occurs in some cases. Oral HPV lesions are typically benign and may resolve without intervention. In all cases, the detection of HPV in a child should prompt careful clinical evaluation to determine the likely transmission route and to assess for the possibility of sexual abuse when clinically indicated.

The forensic implications of pediatric HPV detection remain controversial. While HPV infection has been used as a marker of sexual abuse in some cases, the recognition of multiple non-sexual transmission routes has complicated this interpretation. Current forensic guidelines emphasize that HPV detection alone cannot definitively establish sexual abuse, and that interpretation must consider the child’s age, the anatomical site and characteristics of the lesion, the HPV genotype, the presence of other signs of abuse, and the overall clinical context. In young children with laryngeal or oral HPV, non-sexual transmission is most likely. In older children with anogenital HPV, particularly with high-risk types or with lesions in locations unlikely to result from autoinoculation, sexual abuse becomes a more significant concern [21].

## 4. HPV as a Component of the Human Virome

### 4.1. Metagenomic Evidence

The advent of high-throughput metagenomic sequencing has revolutionized our understanding of the human virome, revealing that HPV is far more prevalent and diverse in healthy populations than previously recognized. Unlike targeted PCR-based assays that detect only specific HPV types included in the assay design, metagenomic approaches provide unbiased detection of all viral sequences present in a sample, enabling discovery of novel types and comprehensive characterization of viral diversity.

Ma and colleagues conducted a landmark metagenomic study of the human virome in healthy adults, using shotgun sequencing to characterize viral communities across multiple body sites [22]. HPV DNA was detected in 68.9% of subjects overall, a prevalence far exceeding that reported in most targeted PCR studies. The study identified 109 distinct HPV types across the cohort, including many types not covered by standard clinical assays. This extraordinary diversity demonstrates that HPV colonization of human epithelial tissues is nearly ubiquitous and involves a much broader range of viral types than those targeted by current diagnostic tests and vaccines [23].

The prevalence of HPV varied substantially across body sites, reflecting the tropism of different viral types for specific epithelial environments. Skin samples showed the highest HPV prevalence at 61.3%, consistent with the known diversity of cutaneous HPV types. Vaginal samples yielded HPV DNA in 41.5% of cases, oral cavity samples in 30%, and gut (stool) samples in 17.3%. These site-specific prevalences indicate that HPV colonization is not limited to the anogenital tract but represents a pan-epithelial phenomenon affecting skin, mucosa, and even the gastrointestinal tract [22].

The metagenomic data also revealed frequent co-infection with multiple HPV types. Many individuals harbored 5–10 or more distinct HPV types simultaneously, with different types showing preferential tropism for different anatomical sites. This multi-type colonization pattern suggests that HPV exists as a complex ecological community within the human virome, with different types occupying distinct niches and potentially interacting through competition, facilitation, or immune modulation.

The clinical implications of these metagenomic findings are profound. The detection of HPV DNA in the majority of healthy adults indicates that HPV presence alone does not equate to disease. Most HPV infections appear to represent asymptomatic colonization or low-level persistent infection that does not progress to dysplasia or cancer. This finding challenges the clinical interpretation of HPV-positive test results, particularly in screening contexts. A positive HPV test may reflect clinically significant infection requiring follow-up, or it may simply detect one component of the normal virome. Distinguishing between these scenarios requires integration of viral load, genotype, persistence over time, and cytological or histological findings.

The metagenomic discovery of 109 HPV types, many not previously characterized, also has implications for vaccine development and disease attribution. Current HPV vaccines target a limited number of high-risk types (primarily 16, 18, 31, 33, 45, 52, and 58 in the nonavalent vaccine) that account for the majority of cervical cancers. However, the existence of dozens of additional types raises questions about the potential for type replacement (where vaccine-targeted types are replaced by non-vaccine types in the ecological niche) and about the disease burden attributable to rare or novel types not currently monitored [22].

### 4.2. HPV in Bodily Fluids

The detection of HPV DNA in diverse bodily fluids beyond genital secretions provides additional evidence for HPV’s status as a ubiquitous virome component and reveals potential transmission routes not previously appreciated. Systematic investigations have documented HPV presence in semen, breast milk, saliva, urine, and blood, though the clinical significance of these findings remains incompletely understood.

Tuominen and colleagues investigated HPV in semen samples from fertile men, detecting viral DNA in 19.4% (6/31) of samples [24]. The presence of HPV in semen has important implications for male reproductive health and for sexual transmission dynamics. Seminal HPV may be transmitted to female partners during intercourse, potentially contributing to cervical infection risk. Additionally, the study found associations between seminal HPV presence and alterations in semen bacterial microbiota, suggesting potential interactions between HPV and the seminal microbiome that could influence fertility or local immune responses [10].

The detection of HPV in breast milk, as discussed previously, was documented by Glenn and colleagues (15% prevalence) and Tuominen and colleagues (8.6% prevalence) [19,20]. The presence of high-risk types HPV-16 and HPV-18 in breast milk samples raises questions about postnatal transmission to nursing infants and potential long-term consequences of early oral exposure to oncogenic HPV types. The mechanisms by which HPV enters breast milk remain unclear but may involve viral shedding from infected ductal epithelium or hematogenous spread [19,20].

HPV DNA has also been detected in saliva and oral rinses from individuals with no visible oral lesions, indicating that oral HPV carriage is common in asymptomatic individuals. The prevalence of oral HPV varies by age, sex, and sexual behavior, with higher rates in men than women and in individuals with greater numbers of oral sex partners. However, oral HPV is also detected in individuals with no history of oral sexual contact, suggesting that non-sexual transmission routes (including vertical transmission, kissing, or environmental exposure) contribute to oral HPV acquisition.

The detection of HPV DNA in blood and serum has been reported in some studies, though the frequency and significance of viremia remain controversial. Unlike viruses that establish systemic infections with sustained viremia (e.g., HIV, hepatitis B), HPV is generally considered a localized epithelial infection. However, transient viremia may occur during active infection, potentially facilitating hematogenous spread to distant sites or transplacental transmission. The detection of HPV DNA in cord blood in some studies supports the possibility of viremia, though the frequency and duration of HPV viremia in natural infections remain poorly characterized. Vergara and colleagues demonstrated the presence of HPV DNA in peripheral blood mononuclear cells (PBMCs) from blood donors, including individuals without known HPV-associated lesions, providing direct evidence that HPV can circulate in the bloodstream in a cell-associated form and may be present in donated blood products [25]. Cocuzza and colleagues similarly detected HPV DNA in plasma samples from women attending a colposcopy clinic, identifying the same genotypes in paired plasma and cervical samples, suggesting that plasma HPV DNA reflects hematogenous dissemination from an active cervical infection rather than mere sample contamination [25]. Together, these findings support the concept that, while HPV is primarily an epitheliotropic virus, viremic episodes do occur and may contribute to extragenital dissemination, transplacental transmission (see Section 2.2), and—in rare circumstances—transfusion-associated infection. The safety implications of HPV viremia for blood transfusion and organ transplantation merit further investigation, particularly for immunocompromised recipients [26].

The presence of HPV in diverse bodily fluids has implications for transmission dynamics and for understanding HPV pathogenesis. Fluids such as semen, saliva, and breast milk represent potential vehicles for viral transmission through sexual contact, kissing, or breastfeeding. The detection of HPV in these fluids also suggests that viral shedding occurs from multiple anatomical sites, not only from visible lesions or dysplastic tissue. This widespread shedding may contribute to the high transmission efficiency of HPV and to the difficulty of preventing transmission through behavioral interventions alone [19,20,24].

### 4.3. Viral Persistence and Stability

The persistence and stability of HPV in human tissues and in the environment represent critical factors determining transmission dynamics and disease risk. Unlike acute viral infections that are rapidly cleared or establish latency in specific cell types, HPV exhibits a complex natural history involving transient infection, persistent infection, latency, and reactivation, with outcomes varying by viral type, anatomical site, and host factors.

Wylie and colleagues conducted longitudinal metagenomic studies of the human virome, documenting the stability of dsDNA viral communities, including papillomaviruses, over time in individuals [16]. The study found that components of the virome, including HPV, can persist stably in individuals over months to years, indicating that many HPV detections represent genuine persistent infections rather than transient contamination or repeated independent acquisitions. This temporal stability supports the concept of HPV as a resident component of the human virome in many individuals [13].

The mechanisms underlying HPV persistence remain incompletely understood but likely involve establishment of viral genomes as episomes in basal epithelial cells, with low-level viral replication that evades immune clearance. In persistent infections, viral gene expression is tightly regulated, with early genes expressed at low levels to maintain the viral genome and late genes (encoding capsid proteins) expressed only in differentiated suprabasal cells. This pattern of gene expression may allow the virus to evade immune surveillance while maintaining a reservoir of infected cells.

The distinction between persistent infection and latency in HPV biology remains controversial. True latency, as defined for herpesviruses, involves maintenance of viral genomes with minimal or no gene expression and no production of infectious virions. Whether HPV establishes true latency or whether all HPV infections involve ongoing low-level replication is debated. Some evidence suggests that HPV can persist in a latent state in basal cells with reactivation triggered by immunosuppression, hormonal changes, or other factors. However, definitive proof of latency requires demonstration of viral genomes in the absence of viral gene expression, which is technically challenging in epithelial tissues with ongoing cell turnover.

The environmental stability of HPV, as discussed in the context of fomite transmission, contributes to the virus’s transmission efficiency. The non-enveloped capsid structure protects viral DNA from environmental degradation, allowing viral particles to remain infectious on surfaces for extended periods. This stability enables transmission through indirect contact with contaminated surfaces and objects, expanding the range of potential transmission routes beyond direct person-to-person contact.

The factors determining clearance versus persistence of HPV infections represent a critical area of ongoing research. Host immune responses, particularly cell-mediated immunity, play a central role in viral clearance. Individuals with robust HPV-specific T cell responses typically clear infections within 1–2 years, while those with impaired cellular immunity (e.g., HIV-infected individuals, transplant recipients) show higher rates of persistent infection and disease progression. Viral factors, including genotype and viral load, also influence persistence, with high-risk types showing greater propensity for persistence than low-risk types.

The persistence of HPV in the human virome has important implications for disease risk and prevention. Persistent infection with high-risk HPV types is the primary risk factor for progression to high-grade dysplasia and cancer. Transient infections, even with high-risk types, rarely progress to disease. Therefore, distinguishing between transient and persistent infections is critical for risk stratification and clinical management. Current screening strategies increasingly incorporate HPV testing with cytology, with repeat testing to identify persistent infections that warrant closer surveillance or intervention [16].

## 5. Clinical and Public Health Implications

### 5.1. Vaccination Strategies

The recognition of non-sexual HPV transmission routes has important implications for vaccination strategies and for optimizing the public health impact of HPV vaccines. Current vaccination programs primarily target pre-adolescent girls and boys before sexual debut, based on the rationale that vaccination before exposure provides maximum protection. However, if non-sexual transmission results in HPV acquisition before adolescence, earlier vaccination or alternative strategies may be warranted.

Population-based vaccination programs have demonstrated substantial real-world effectiveness in reducing HPV-related disease. Petca and colleagues reviewed evidence from national vaccination programs, documenting measurable declines in high-grade cervical abnormalities, genital warts, and HPV prevalence in vaccinated cohorts compared to unvaccinated cohorts [7]. These successes validate the current approach of targeting adolescents before sexual debut. However, the review also noted that non-sexual transmission routes may limit vaccine effectiveness if individuals are exposed to HPV before vaccination [14].

The potential for vertical transmission to establish persistent infection before vaccination age raises questions about optimal vaccination timing. If a substantial proportion of individuals acquire HPV through vertical transmission in infancy, with persistence into childhood, then vaccination at age 11–12 (as currently recommended in many countries) may miss an opportunity to prevent these early-life infections. However, the data from Park and colleagues showing 100% clearance of neonatal HPV by 6 months, and from Hijona Elosegui and colleagues showing 94.3% clearance of oropharyngeal colonization by 24 months, suggest that most vertically transmitted infections clear spontaneously before vaccination age [1,10]. Therefore, earlier vaccination (e.g., in infancy) may not provide substantial additional benefit for the majority of individuals.

An important limitation of current vaccines is their restricted genotype coverage. The nonavalent vaccine targets nine HPV types (6, 11, 16, 18, 31, 33, 45, 52, 58), which account for approximately 90% of cervical cancers and 90% of genital warts. However, the metagenomic data from Ma and colleagues documenting 109 HPV types in healthy individuals indicates that the majority of HPV diversity is not covered by current vaccines [22]. While most non-vaccine types are not associated with cancer, the potential for type replacement or for disease burden from rare types remains a concern for long-term vaccine impact.

Smith and colleagues examined vertical transmission pairs and noted that the HPV types transmitted from mothers to infants in their cohort were not among the vaccine-covered types [6]. This observation led the authors to conclude that maternal vaccination before pregnancy would be unlikely to prevent the specific vertical transmissions observed in their study. However, this conclusion must be interpreted cautiously, as the study was small and may not be representative of all vertical transmission events. Larger studies with more comprehensive genotyping are needed to determine what proportion of vertical transmissions involve vaccine-preventable types [9].

Vaccination of pregnant women to prevent vertical transmission represents a potential strategy that has not been extensively evaluated. Maternal vaccination could theoretically boost antibody levels that are transferred transplacentally and through breast milk, providing passive immunity to the infant during the period of highest vertical transmission risk. However, HPV vaccines are not currently recommended during pregnancy due to limited safety data, and the potential benefit of maternal vaccination for preventing vertical transmission remains speculative.

The public health implications of non-sexual transmission for vaccination policy include the need for continued surveillance of HPV prevalence and disease burden in vaccinated populations, monitoring for potential type replacement, and evaluation of whether earlier vaccination or broader genotype coverage would provide additional benefit. Education of healthcare providers and the public about non-sexual transmission routes is also important to address concerns about HPV detection in children and non-sexually active individuals and to promote vaccine acceptance [7].

### 5.2. Screening and Test Interpretation

The recognition that HPV is a ubiquitous virome component detected in the majority of healthy adults has profound implications for the interpretation of HPV test results in screening and clinical contexts. The challenge lies in distinguishing between clinically significant persistent infections that warrant intervention and low-level viral carriage that represents a component of the normal virome without disease risk.

Current cervical cancer screening guidelines in many countries incorporate HPV testing, either as primary screening or as co-testing with cytology. A positive HPV test typically triggers reflex cytology, repeat testing, or colposcopy, depending on the specific algorithm and the patient’s age and screening history. However, the metagenomic data from Ma and colleagues showing HPV DNA in 68.9% of healthy adults raises questions about the specificity of HPV testing [22]. If the majority of individuals harbor HPV DNA at some body site, then detection of HPV in cervical samples may not always indicate clinically significant infection.

Several factors help distinguish clinically significant infections from benign viral carriage. Viral load is a critical determinant, with high viral loads indicating active replication and greater disease risk. Most clinical HPV assays are qualitative (positive/negative) rather than quantitative, but quantitative assays that measure viral load may provide better risk stratification. Genotype is also important, with high-risk types (particularly 16 and 18) conferring substantially greater cancer risk than low-risk types. Persistence over time is perhaps the most important factor, as persistent infection with high-risk types is the primary driver of progression to high-grade dysplasia and cancer.

Current screening algorithms attempt to incorporate these factors through repeat testing and integration with cytology. For example, women with a positive HPV test but negative cytology may be retested in 1 year, with persistent HPV positivity triggering colposcopy. This approach identifies individuals with persistent infections while allowing transient infections to clear without intervention. However, the optimal algorithms for integrating HPV testing, cytology, and clinical management continue to evolve as more data become available.

The detection of HPV in non-cervical sites (oral cavity, skin, anus) presents additional interpretive challenges. Oral HPV testing is not currently recommended for routine screening, despite the increasing incidence of HPV-associated oropharyngeal cancer. The high prevalence of oral HPV in healthy individuals (30% in the Ma study) and the low positive predictive value of oral HPV testing for cancer risk make routine screening impractical with current technology [22]. Similarly, anal HPV testing is recommended for high-risk populations (e.g., HIV-infected individuals, men who have sex with men) but not for the general population.

The interpretation of HPV test results in children and non-sexually active individuals requires particular caution. As discussed previously, HPV detection in these populations may result from vertical transmission, fomite exposure, or horizontal non-sexual contact rather than sexual activity. Clinicians must avoid inappropriate conclusions about sexual activity or abuse based solely on HPV detection. In pediatric cases, HPV testing is generally not recommended unless there is a clinical indication (e.g., visible lesions), and positive results should be interpreted in the context of the child’s age, the anatomical site, and the overall clinical picture.

The widespread detection of HPV in metagenomic studies also has implications for research and for the development of new diagnostic approaches. Future assays may need to incorporate viral load measurement, genotyping for multiple high-risk types, and assessment of viral integration status (as integrated HPV genomes are associated with higher cancer risk than episomal genomes). Biomarkers of viral activity, such as E6/E7 mRNA expression or p16 immunostaining, may provide better indicators of clinically significant infection than DNA detection alone [16,22].

### 5.3. Infection Control in Healthcare Settings

The environmental stability of HPV and its detection on medical devices and surfaces in healthcare settings necessitate rigorous infection control measures to prevent nosocomial transmission. Standard cleaning and disinfection protocols used in many healthcare facilities may be inadequate for complete HPV inactivation, particularly for instruments with complex geometries or porous materials.

Ryndock and Meyers conducted a comprehensive review of HPV environmental contamination and infection control challenges [17]. HPV DNA has been detected on vaginal specula, colposcopes, examination tables, and other equipment in gynecology clinics, even after standard cleaning procedures. The virus’s non-enveloped capsid structure confers resistance to many disinfectants that are effective against enveloped viruses. High-level disinfection or sterilization is required for complete viral inactivation, but many reusable instruments in gynecology and other specialties receive only low-level or intermediate-level disinfection [4].

Sabeena and colleagues conducted systematic investigations of HPV contamination in healthcare settings, identifying viral DNA on reusable medical instruments and environmental surfaces [18]. The potential for patient-to-patient transmission via inadequately disinfected instruments represents a serious infection control concern. The authors recommend enhanced disinfection protocols, including use of high-level disinfectants or sterilization for instruments that contact mucous membranes, implementation of single-use disposable instruments where feasible, and regular environmental surface disinfection in examination rooms [5].

Specific recommendations for infection control in healthcare settings include:

1. Instrument reprocessing: Reusable instruments that contact mucous membranes (e.g., vaginal specula, anoscopes) should undergo high-level disinfection or sterilization between patients. Instruments with complex geometries that are difficult to clean should be replaced with single-use disposable alternatives where possible.
2. Environmental surface disinfection: Examination tables, stirrups, and other surfaces that may be contaminated with genital secretions should be cleaned and disinfected between patients using EPA-registered disinfectants with virucidal activity against non-enveloped viruses.
3. Hand hygiene: Healthcare providers should perform hand hygiene before and after patient contact, as HPV can be transferred on hands and gloves. Gloves should be changed between patients and should not be reused.
4. Barrier methods: For equipment that cannot be adequately disinfected (e.g., certain colposcopes, ultrasound transducers), disposable barrier covers should be used and changed between patients.
5. Education and training: Healthcare personnel should receive education about HPV transmission routes, environmental stability, and proper infection control procedures. Compliance with infection control protocols should be monitored and reinforced.

The cost-effectiveness of enhanced infection control measures for HPV prevention has not been formally evaluated. However, given the high prevalence of HPV, the potential for nosocomial transmission, and the serious consequences of HPV-related disease, investment in improved infection control is likely justified. The increasing recognition of non-sexual transmission routes, including nosocomial transmission, should prompt healthcare facilities to re-evaluate their current disinfection protocols and to implement enhanced measures where indicated [17,18].

### 5.4. Pediatric Follow-Up and Counseling

The detection of HPV in children presents unique clinical and counseling challenges. As discussed previously, most neonatal and early-life HPV detections represent transient colonization that clears spontaneously without intervention. However, a subset of infections persist and may lead to clinical disease, necessitating careful follow-up and appropriate counseling of parents and caregivers.

For neonates born to HPV-positive mothers, particularly those with high-risk types or types 6 and 11 (associated with respiratory papillomatosis), clinical surveillance during the first years of life may be warranted. Parents should be educated about signs and symptoms of respiratory papillomatosis, including hoarseness, stridor, noisy breathing, or respiratory distress. Early recognition and referral to otolaryngology for evaluation and management can prevent serious complications. However, routine HPV testing of asymptomatic neonates is not recommended, as the high rate of spontaneous clearance and the lack of effective preventive interventions make screening impractical [1,10].

For children with detected HPV infections or HPV-associated lesions, counseling should address the likely transmission route, the natural history of the infection, and the implications for the child’s health and for family members. In young children (particularly those under age 3–4), parents should be reassured that non-sexual transmission routes (vertical, fomite, horizontal contact) are most likely, and that HPV detection does not indicate sexual activity or abuse. In older children, particularly those with anogenital lesions, the possibility of sexual abuse must be considered, and appropriate evaluation and referral to child protective services may be indicated based on the overall clinical context [21].

The management of pediatric HPV infections depends on the anatomical site and clinical presentation. Laryngeal papillomatosis requires surgical intervention, often with multiple procedures over years, and may benefit from adjuvant therapies such as intralesional cidofovir or systemic bevacizumab in severe cases. Anogenital warts may be treated with topical therapies (imiquimod, podophyllotoxin) or surgical removal, though spontaneous regression occurs in some cases. Oral lesions are typically benign and may be observed without intervention unless they cause symptoms or cosmetic concerns.

Counseling of parents should also address prevention of transmission to siblings or other household members. While the risk of horizontal transmission within households appears to be low, basic hygiene measures (e.g., not sharing towels or bathing implements, hand hygiene after diaper changes) may reduce transmission risk. Parents should be reassured that routine household contact (hugging, kissing on the cheek, sharing meals) does not pose significant transmission risk and that isolation of the affected child is not necessary.

For adolescents with HPV detection, counseling should address both non-sexual and sexual transmission routes. Adolescents who are not yet sexually active should be educated about the possibility of non-sexual acquisition and reassured that HPV detection does not necessarily indicate sexual activity. For sexually active adolescents, counseling should include information about sexual transmission, the importance of vaccination (if not already vaccinated), and strategies to reduce transmission risk. However, clinicians should emphasize that HPV is extremely common, that most infections clear spontaneously, and that HPV detection should not be stigmatized.

The psychological impact of HPV diagnosis in children and adolescents should not be underestimated. Parents may experience guilt, anxiety, or confusion about how their child acquired the infection. Adolescents may experience stigma, anxiety about cancer risk, or concerns about disclosure to partners. Clinicians should provide clear, accurate information about transmission routes, natural history, and prognosis, and should address emotional and psychological concerns as part of comprehensive care [21].

## 6. Conclusions

The accumulating evidence reviewed in this article demonstrates that HPV transmission extends far beyond sexual contact, encompassing vertical transmission from mother to child, environmental contamination and fomite transmission, horizontal non-sexual contact, and potentially respiratory routes. The detection of HPV DNA in neonates, infants, children, and female virgins—populations with no possibility of sexual transmission—provides compelling evidence for these alternative acquisition routes. Furthermore, metagenomic studies reveal that HPV is a ubiquitous component of the human virome, with viral DNA detected in the majority of healthy adults across multiple body sites and encompassing extraordinary viral diversity far exceeding the types targeted by current diagnostic assays and vaccines.

Vertical transmission occurs in approximately 18.2% of HPV-positive mothers, with neonatal colonization rates reaching 58.2% for oropharyngeal sites following vaginal delivery. While most vertically transmitted infections clear spontaneously within the first 6–24 months of life, a small subset persist and may lead to clinical disease, particularly juvenile-onset recurrent respiratory papillomatosis. Transplacental transmission, documented through detection of HPV DNA in placental tissue and cord blood with mother–placenta–newborn genotype concordance, represents an additional route of vertical transmission that may establish in utero infection.

The environmental stability of HPV enables transmission through contaminated surfaces, medical devices, and fomites. HPV DNA has been detected on medical instruments in healthcare settings even after standard cleaning procedures, raising concerns about nosocomial transmission and highlighting the need for enhanced infection control protocols. The detection of HPV in breast milk (8.6–15% prevalence) and the high prevalence of oral HPV in nursing infants (40%) suggest that breastfeeding may represent an additional postnatal transmission route, though the clinical significance of these exposures remains uncertain.

Metagenomic evidence positions HPV as a core component of the human virome rather than solely a pathogenic infection. HPV DNA is detected in 68.9% of healthy adults, with 109 distinct types identified across body sites including skin (61.3%), vagina (41.5%), oral cavity (30%), and gut (17.3%). This widespread colonization indicates that HPV presence alone does not equate to disease, and that most HPV infections represent asymptomatic carriage or low-level persistent infection without progression to dysplasia or cancer. The challenge for clinical practice lies in distinguishing between clinically significant persistent infections with high-risk types that warrant intervention and benign viral carriage that represents a component of the normal virome.

These findings have important implications for clinical practice and public health policy. Vaccination strategies must account for the possibility of non-sexual transmission, though current evidence suggests that most vertically transmitted infections clear before vaccination age and that vaccination of pre-adolescents before sexual debut remains the optimal strategy. Screening and diagnostic test interpretation must consider that HPV detection, particularly at low viral loads or with non-high-risk types, may reflect virome components rather than clinically significant infection. Integration of viral load, genotype, persistence over time, and cytological findings is essential for appropriate risk stratification.

Infection control in healthcare settings requires enhanced attention to disinfection protocols, with high-level disinfection or sterilization of reusable instruments that contact mucous membranes, use of single-use disposable instruments where feasible, and regular environmental surface disinfection. Healthcare providers should receive education about non-sexual transmission routes and proper infection control procedures to minimize nosocomial transmission risk.

Pediatric follow-up and counseling must recognize that HPV detection in children most commonly results from non-sexual transmission routes, particularly in young children. While most neonatal and early-life infections clear spontaneously, surveillance for signs of respiratory papillomatosis in high-risk infants and appropriate management of persistent infections or clinical lesions is warranted. In older children with anogenital lesions, careful evaluation for possible sexual abuse is indicated, but HPV detection alone cannot definitively establish abuse.

Critical knowledge gaps remain that warrant further investigation. Standardized longitudinal studies with sensitive, type-specific assays are needed to resolve discordant prevalence estimates across studies and to clarify the natural history of infections acquired through different routes. The factors determining clearance versus persistence of vertically transmitted and other non-sexually acquired infections require elucidation to enable risk stratification and targeted interventions. The clinical significance of HPV detection in breast milk and the long-term consequences of early oral exposure to high-risk types merit further study. The potential for type replacement following widespread vaccination and the disease burden attributable to non-vaccine types detected in metagenomic studies should be monitored through ongoing surveillance.

A research roadmap for the field of non-sexual HPV transmission should prioritize: (i) development and harmonization of ultra-sensitive, type-specific assays for HPV quantification across non-genital matrices (blood, breast milk, oral secretions, fomites); (ii) large-scale prospective mother–infant cohort studies with serial sampling from pregnancy through early childhood to resolve the natural history of perinatally acquired infections; (iii) mechanistic studies interrogating the role of host immune ontogeny, HLA haplotype, and mucosal microbiome composition in determining clearance versus persistence of non-sexually acquired HPV; (iv) epidemiological surveillance of JORRP incidence across populations with differential HPV vaccination coverage to quantify vaccine-preventable burden [12,15]; (v) metagenomic investigation of whether HPV virome diversity has functional consequences for mucosal immunity and susceptibility to oncogenic HPV types [2,3]; and (vi) evaluation of expanded vaccination strategies—including maternal immunization and gender-neutral school-based programs—for reducing non-sexual HPV transmission and its clinical sequelae [13].

In conclusion, HPV should be recognized as a ubiquitous virome component with multiple acquisition routes extending beyond sexual transmission. This expanded understanding of HPV epidemiology has important implications for clinical interpretation of test results, for counseling of patients and families, for infection control practices, and for public health strategies. While sexual transmission remains the primary route for acquisition of high-risk types associated with cancer, the recognition of non-sexual transmission routes is essential for comprehensive understanding of HPV natural history and for optimal clinical management across all age groups and populations.

## Data Availability

No new data were created or analyzed in this study. Data sharing is not applicable to this review article.

## References

[1] Park J.S., Ryu H.S., Kim J.Y., Choi H.J., Nam S.Y., Kim J.H., Chung S.Y., Park S.Y. (2012). Vertical transmission of human papillomavirus from mothers to infants: Relationship between infection rate and mode of delivery. Virol. J..

[2] Rombaldi R.L., Serafini E.P., Mandelli J., Zimmermann E., Losquiavo K.P. (2008). Transplacental transmission of human papillomavirus. Virol. J..

[3] Elosegui J.J.H., Gómez M.Á.S., Hernández M.S., Gordo J.M.A. (2022). Oropharyngeal colonization by human papillomavirus in newborns: A prospective follow-up study. J. Clin. Virol..

[4] Ryndock E.J., Meyers C. (2014). A risk for non-sexual transmission of human papillomavirus?. Expert Rev. Anti-Infect. Ther..

[5] Sabeena S., Bhat P., Kamath V., Arunkumar G. (2017). Possible non-sexual modes of transmission of human papilloma virus. J. Obstet. Gynaecol. Res..

[6] Ardekani A., Gharib M., Seyedhossaini S., Amini S., Razaviyan J., Ghasemian A., Javadinia S.A., Rezaie F. (2022). Human Papillomavirus Infection during Pregnancy and Childhood: A Comprehensive Review. Microorganisms.

[7] Sinclair K.A., Woods S.A., Kirse B.J. (2005). Anogenital and respiratory tract human papillomavirus infections among children: Age, gender, and potential transmission through sexual abuse. Pediatrics.

[8] Koskimaa H.M., Paaso A.E., Welters M.J., Grénman S.E., Syrjänen K.J., van der Burg S.H., Syrjänen S.M. (2017). The presence of human papillomavirus (HPV) in placenta and/or cord blood might result in T-cell mediated responses in the neonate. Eur. J. Clin. Microbiol. Infect. Dis..

[9] Smith E.M., Ritchie J.M., Yankowitz J., Swarnavel L., Wang L.P., Haugen D., Turek L.P. (2004). Human papillomavirus prevalence and types in newborns and parents: Concordance and modes of transmission. Sex. Transm. Dis..

[10] Tuominen H., Rautava S., Syrjänen S., Collado S., Syrjänen K. (2021). HPV infection and bacterial microbiota in semen of men attending fertility clinic. Microorganisms.

[11] Glenn W.K., Whitaker C.H., Lawson J.A. (2012). High risk human papillomavirus and Epstein Barr virus in human breast milk. BMC Res. Notes.

[12] Tuominen H., Rautava S., Collado M., Syrjänen S., Syrjänen K. (2018). HPV infection and bacterial microbiota in breast milk and infant oral mucosa. PLoS ONE.

[13] Wylie K.M., Mihindukulasuriya K.A., Zhou Y., Sodergren E., Weinstock G.M., Storch G.A. (2014). Metagenomic analysis of double-stranded DNA viruses in healthy adults. BMC Biol..

[14] Petca A., Petca R.C., Miulescu M., Dumitrascu C.E., Georgescu S.R., Ionescu N., Sandru C., Petca F. (2020). Non-sexual HPV transmission and role of vaccination for a better future (Review). Exp. Ther. Med..

[15] Duan Y., Wu Y., Li Y., Zhang H., Sun Y., Li M., Jia S. (2025). Monitoring the presence of human papillomavirus in public areas indicates potential non-sexual transmission routes. BMC Public Health.

[16] Bertinazzi M., Ercoli G., Biasini A., Ramponi A., Lacroix C., Guani B., Monnier-Cholley L., Mondain M., Durdux C., Baujat B. (2022). Clinical implications of alpha, beta and gamma human papillomavirus in juvenile-onset recurrent respiratory papillomatosis. Eur. Arch. Otorhinolaryngol..

[17] Sasivimolrattana T., Chuenchor W., Manuyakorn W., Bhakdisongkhram P., Payungporn S., Poovorawan Y., Hirankarn N., Palaga T. (2022). Human Virome in Cervix Controlled by Domination of Human Papillomavirus. Viruses.

[18] Chatzistamatiou K., Sotiriadis A., Agorastos T. (2016). Effect of mode of delivery on vertical human papillomavirus transmission—A meta-analysis. J. Obstet. Gynaecol..

[19] Lépine M., Beauchamp M.C., Boulanger M.C., Tétreault A., Phan T.A. (2024). Juvenile Onset Recurrent Respiratory Papillomatosis: What do we know in 2024?. Tumour Virus Res..

[20] Jacobsen L.M., Österberg E.C., Koch A., Buchwald C. (2024). Trends in the incidence and prevalence of juvenile-onset recurrent respiratory papillomatosis in Denmark. Acta Otolaryngol..

[21] Amiling R., Forrest C.B., Feudtner C., Mollen C., Gerber J.S. (2021). Juvenile-Onset Recurrent Respiratory Papillomatosis in the United States: An Epidemiologic Study. J. Pediatr. Infect. Dis. Soc..

[22] Park J., Sedaghat A.R., Ching R., Clavijo L.C., Noonan K.Y. (2022). Systematic review of the use of the human papillomavirus vaccine as adjuvant therapy for juvenile-onset recurrent respiratory papillomatosis. Int. J. Pediatr. Otorhinolaryngol..

[23] Ma Y., Madupu L., Karaoz K., Belcaid A., Podar P.W., Laver S., Huang L., Hugenholtz P., Ivanova E., Nelson K.E. (2014). Human papillomavirus community in healthy persons, defined by metagenomics analysis of human microbiome project shotgun sequencing data sets. J. Virol..

[24] Vergara C., Marrades R., Pellegrini C., Ballester O., González-López J.J., Prat J., Ramírez J. (2019). Detection and quantitation of human papillomavirus DNA in peripheral blood mononuclear cells from blood donors. J. Med. Virol..

[25] Cocuzza C.E., Serioli M., Galli L., Morosi S., Semino M., Pisani G., Sarasini A., Baldanti F., Principi N., Esposito S. (2017). Human papillomavirus DNA detection in plasma and cervical samples of women attending a colposcopy clinic. PLoS ONE.

[26] Ardekani A., Daliran A., Talari H., Ahadinia E., Hoseini M.J., Rahnama M., Goudarzi Yeganeh M. (2022). Worldwide prevalence of human papillomavirus among pregnant women: A systematic review and meta-analysis. Rev. Med. Virol..

